# Role of LGMN in tumor development and its progression and connection with the tumor microenvironment

**DOI:** 10.3389/fmolb.2023.1121964

**Published:** 2023-02-07

**Authors:** Safir Ullah Khan, Ibrar Muhammad Khan, Munir Ullah Khan, Muhammad Azhar Ud Din, Muhammad Zahoor Khan, Nazir Muhammad Khan, Yong Liu

**Affiliations:** ^1^ Anhui Province Key Laboratory of Embryo Development and Reproduction Regulation, Anhui Province Key Laboratory of Environmental Hormone and Reproduction, School of Biological and Food Engineering, Fuyang Normal University, Fuyang, China; ^2^ Hefei National Laboratory for Physical Sciences at the Microscale, School of Life Sciences, University of Science and Technology of China, Hefei, China; ^3^ MOE Key Laboratory of Macromolecular Synthesis and Functionalization, Department of Polymer Science and Engineering, International Research Center for X Polymers, Zhejiang University, Hangzhou, China; ^4^ Faculty of Pharmacy, Gomal University Dera Ismail Khan KPK, Dera IsmailKhan, Pakistan; ^5^ Department of Animal Breeding and Genetics, Faculty of Veterinary and Animal Sciences, University of Agriculture, Dera IsmailKhan, Pakistan; ^6^ Department of Zoology, University of Science and Technology, Bannu, Pakistan

**Keywords:** legumain, asparagine endopeptidase, tumor microenvironment, biological mechanism, glioblastoma, gastric cancer

## Abstract

Legumain (LGMN) has been demonstrated to be overexpressed not just in breast, prostatic, and liver tumor cells, but also in the macrophages that compose the tumor microenvironment. This supports the idea that LGMN is a pivotal protein in regulating tumor development, invasion, and dissemination. Targeting LGMN with siRNA or chemotherapeutic medicines and peptides can suppress cancer cell proliferation in culture and reduce tumor growth *in vivo*. Furthermore, legumain can be used as a marker for cancer detection and targeting due to its expression being significantly lower in normal cells compared to tumors or tumor-associated macrophages (TAMs). Tumor formation is influenced by aberrant expression of proteins and alterations in cellular architecture, but the tumor microenvironment is a crucial deciding factor. Legumain (LGMN) is an in vivo-active cysteine protease that catalyzes the degradation of numerous proteins. Its precise biological mechanism encompasses a number of routes, including effects on tumor-associated macrophage and neovascular endothelium in the tumor microenvironment. The purpose of this work is to establish a rationale for thoroughly investigating the function of LGMN in the tumor microenvironment and discovering novel tumor early diagnosis markers and therapeutic targets by reviewing the function of LGMN in tumor genesis and progression and its relationship with tumor milieu.

## 1 Introduction

Legumain (LGMN) is an asparaginyl-specific cysteine endopeptidase that is a member of the peptidase family C13 (Barrett and Rawlings, 1996). Legumain was first discovered in plants3, while in mammals, a glycoprotein with a 34-kilodalton molecular weight (LGMN) was first identified in pigs (Chen et al., 1997). The gene has been around for a long time and has been shown to be expressed in a variety of organisms, including mammals and humans (Kembhavi et al., 1993; Liu et al., 2003). The proximal tubule is the primary site of LGMN distribution in mouse kidneys (Yamane et al., 2002). The majority of lysosome-localized legumain is expressed, but the protein is also found in the periplasm and the cytoplasm (Haugen et al., 2015). In contrast, 13%–17% of the entire LGMN is localized to the nucleus in colon cancer cell lines. Specifically, legumain expression in malignancies has been the subject of a growing number of research efforts.

One of the leading causes of death among people is cancer. World Health Organization (WHO) estimates that by 2020, cancer will have caused the deaths of roughly 10 million people worldwide (Sung et al., 2021; Gao et al., 2022). As a result of individuals being detected with cancer at an earlier stage, the rate at which it can be cured will considerably increase. Due to the importance of early diagnosis in increasing both the cure rate and the survival rate of patients, the ability to quickly and easily identify a variety of biomarkers in organisms is of paramount importance (Singal et al., 2017). Selecting disease-specific biomarkers is crucial for accurate cancer diagnosis and localization (Chen et al., 2018). Since enzymes are involved in nearly every biological process and accurately represent the state of the organism with respect to cancer, they have emerged as a promising signal for the detection of the disease at an early stage (Lin et al., 2016).

The cysteine protease known as legumain is most commonly located in lysosomes, which are located in highly acidic (Liu et al., 2003; Edgington et al., 2013; Mai et al., 2017; Mathur et al., 2020). Legumain up-regulation is associated with tumor invasion, proliferation, and angiogenesis (Lin et al., 2020a). Multiple cancer types, including breast, colorectal (Zhao et al., 2018), and gastric, have been linked to the overexpression of legume proteases at high levels (Gawenda et al., 2007; Liu et al., 2014; Yamane et al., 2017; He et al., 2018). Because legume proteases play a role in the formation of tumors, medical professionals consider them to be significant biomarkers for the diagnosis of cancer. A precise assessment of legume proteolytic activity and a precise localisation of tumors are crucial for early cancer diagnosis, as it is believed that cancer can be cured in its early stages (Harbeck, 2018; Romeo et al., 2022).

Legumain (LGMN) is also called peptide enzyme in asparagine (asparagine endopeptidase, AEP), as early as the 20th century in leguminous seeds, was found in the early 80 s and 1997, for the first time in mammals is separated identification (Csoma and Polgár, 1984; Chen et al., 1997), It is the only known cysteine protease that cuts the peptide bond of asparagine residues explicitly in the mammalian genome and belongs to a new member of cysteine protease C13 family (Zhang and Lin, 2021). Breast cancer, stomach cancer, and other malignancies have all been linked to LGMN expression and distribution (Murthy et al., 2005; Wu et al., 2006). LGMN can influence tumor cell growth, propagation, and migration by reshaping extracellular substrates, modulating exosome-mediated chemical transport, and impacting the angiogenesis of the tumor microenvironment (Shen et al., 2016; Yan et al., 2018; Li et al., 2020). Recent years have brought a lot of attention to LGMN and its function in the tumor microenvironment. This work aims to encourage research into LGMN’s mechanism of action in cancer development by reviewing its phenotypic characteristics in malignant tumor tissues, its method of action in tumor development, and its regulatory effect on the tumor microenvironment.

## 2 Biological characteristics of LGMN

LGM is localized in human 14q32.1 and consists of 14 exons and 13 introns, encoding a polypeptide chain composed of 433 amino acid residues. It was showed through a co-immunoprecipitation experiment that there were three forms of human LGMN protein, namely 56 kDa progenitor, 46 and 36 kDa mature enzyme, among which the progenitor was not active (Lewēn et al., 2008). By adjusting the pH in the environment, the autocatalytic process can be initiated to transform the enzyme into a functional 46 kDa mature enzyme. After various proteases treated the 46 kDa mature enzyme in lysozyme, the carboxyl terminal of the 46 kDa mature enzyme was cleaved to produce 36 kDa mature enzyme, and the activities of the two forms of enzyme were consistent. The structure of LGMN precursor is mainly composed of three parts: signal peptide, catalytic functional region, and legumain stabilization and activity modulation (LSAM). The signal peptide partially guides LGMN to the endoplasmic reticulum for processing. The catalytic functional region stabilizes the enzyme activity of LGMN through the three active site residues, His148, Cys189, and Asn42 which are close to each other in space. LSAM ensures the stable state of the LGMN precursor by triggering the electrostatic coded stabilization switch near the catalytic structure domain (Dall and Brandstetter, 2013; Zhao et al., 2014; Reddy et al., 2021).

LGMN is mainly distributed in lysosomes and mediates the processing of various kinds of albumin, such as the conversion of cysteine cathepsin from single-stranded to double-stranded form. It also plays a vital role in mammalian immunity. B cells and dendritic cells are two examples of antigen-presenting cells seen in mammals that express LGMN. Tolerance to immunological stimulation can be fostered because LGMN is processed in B cells along with foreign proteins to display Class II major histocompatibility complexes on the membrane of T cells (Manoury et al., 1998; Trombetta et al., 2003). Toll-like receptors (TLRs) are involved in innate immune responses and are activated by cathepsin and other similar receptors. LGMN is involved in normal biological processes, but it has also been linked to several pathologies, including cancer. Several types of solid tumors, including those of the breast, colon (Haugen et al., 2013), prostrate (Ohno et al., 2013), and ovary (Wang et al., 2012), have been found to express LGMN at high levels in tumor neovascular endothelial cells, TAMs inside the tumor stroma, and in the tumors themselves. Its symptoms correlate highly with clinical markers and prognosis for a wide range of tumor types. Additionally, several cells inside the tumor microenvironment showed signs of having high LGMN expression (Reisfeld, 2013).

## 3 The relationship between LGMN and tumor

LGMN is a protein that is significantly expressed in a number of tumor disorders, including ovarian, breast, and cervical cancer. Its expression level varies depending on the patient’s clinical status or tumor grade. The poor clinical stage is indicated by the high expression level. It was used an immunohistochemical approach to examine 84 cases of standard cervical cancer samples, 62 cases of precancerous lesions, and 41 cases of cervical cancer (Boon et al., 2022). They verified that LGMN expression levels in cervical tumor tissue were much greater than those found in premalignant lesions and healthy tissues.

Similarly, It was found through immune histochemistry that the higher the expression of LGMN in breast cancer, the higher the tumor grade (Toss et al., 2019). In addition, some scholars verified the positive correlation between the increase of LGMN expression and the malignancy degree of ovarian tumors by real-time PCR and western blot. These correlations suggest that LGMN may be used as a screening and auxiliary diagnostic indicator for malignant tumors.

Malignant tumor cells’ invasion and metastasis can be aided by LGMN. After injecting mice with LGMN protein, It was discovered that the protein promoted the proliferation and spread of breast cancer cells (Xu et al., 2017). The LGMN AEPI (Aza-Asn epoxides significantly block the activity of AEP) inhibitors, on the other hand, confirmed that the LGMN protein might influence the expansion and migration of breast cancer cells. In a similar manner, HeLa and SiHa cells have the LGMN gene knocked out (Meng and Liu, 2016). By observing a marked decrease in its migratory and invasion capacity, they concluded that LGMN played a critical part in these processes in cervical cancer cells. LGMN over expression has been shown to increase tumor cell migration and invasion *in vitro* using cell lines derived from ovarian and stomach cancers (Wang et al., 2020).

In addition to being linked to advanced disease and the spread of cancer, LGMN over expression also has a negative impact on patient outcomes. From their research following patients with ovarian cancer, It was concluded that those with high LGMN expression were more likely to have a poor outcome than those with low expression. Similar results were seen by monitoring 73 patients with stomach cancer and discovered a negative connection between LGMN expression and cumulative survival (Li et al., 2013; Lunde et al., 2017). High LGMN expression in numerous malignant tumor cells has been found to alter prognosis (Lin et al., 2014; Poreba et al., 2016), and several studies have demonstrated that patients with high LGMN expression have a lower relative survival.

## 4 Mechanism of LGMN in tumor formation and progression

With the development of molecular biology techniques, the mechanism of LGMN’s involvement in tumor occurrence and development has been gradually studied and revealed, such as exosome transport, regulation of protease activity, regulation of integrin interaction, signaling pathway intervention, *etc.* (Table 1).

**TABLE 1:** Mechanism of LGMN in tumor formation and progression.

Signaling pathway	Signal change	Tumor categories	Effect	References
LGMN/PI3K/AKT	initiation	Breast cancer	Induce breast cancer cell spreading and invasion	Cui et al. (2016)
LGMN/TGF-β	initiation		Repolarize macrophages linked with tumors	Jin et al. (2019)
LGMN/MMP-2	initiation	Breast cancer	encourage breast cancer cells to spread and invade	Yun et al. (2022)
LGMN, Integrin	Inhibition	Breast cancer	Incite breast cancer cell spreading	Gao et al. (2013), Jailani et al. (2022)
MMP-9	Activation	Gastric cancer	Accelerate the spread and invasion of stomach cancer	Yu et al. (2021)
miR-3978	Inhibition	Gastric Carcinoma	Accelerate the spread and invasion of stomach cancer	Li et al. (2013)
P53	Inhibition	Glioblastoma	Induce cancer cell spreading and invasion	Peng et al. (2018)
integrin α5β1	Coactivation	Epithelial Ovarian	Repolarize macrophages linked with tumors	Poste and Fidler (1980)
		Carcinoma		
integrin αvβ3	Inhibition	Breast Carcinoma	encourage breast cancer cells to spread and invade	Mai et al. (2017)
Pro-MMP-2, -9	Activation	Breast cancer	Induce breast cancer cell spreading and invasion	Huang et al. (2017), Kang et al. (2019)
LGMN, Integrin	Co-activation	Epithelial ovarian cancer	α5β1/AEP complex affects epithelial ovarian cancer proliferation and migration	Quail and Joyce (2013)

### 4.1 LGMN participates in MMP-2 and MMP-9 signaling mechanism

The extracellular matrix is broken down and remodeled by matrix metalloproteinases (MMPS), zinc-dependent endopeptidases that are essential components of the tumor microenvironment. The MMP family’s MMP-2 and MMP-9 will develop enzyme activity as a result of the cleavage of LGMN (Chen et al., 2001). Gelatin genes’ N-terminal asparagine bonds are broken by activated LGMN, resulting in the maturation of MMP-2 and MMP-9. Both facilitate breast cancer cells’ invasion and metastasis and contribute to the breakdown of the extracellular matrix (Kang et al., 2019). LGMN either directly or indirectly affects MMP-9 in gastric cancer, causing it to gradually change from an inactive protease form into an active MMP-9 form that inhibits the activity of the extracellular matrix and encourages the spread of gastric cancer cells (Tu et al., 2022).

### 4.2 LGMN participates in the PI3K/AKT signaling pathway

AKT is a serine/threonine kinase, while PI3K is a phosphatidylinositol 3-hydroxykinase. The PI3K/AKT pathway controls the invasion, metastasis, and proliferation of cells. By preventing cell apoptosis and fortifying resistance to chemotherapy-induced apoptosis, phosphoinositol 3-kinase accelerates the growth of tumors. This increases the capacity of breast cancer cells to invade and metastasize. Epithelial-mesenchymal transitions (EMT) also encourage the invasion and metastasis of breast cancer (Rafael et al., 2015; Thapa et al., 2015; Dirican and Akkiprik, 2017; Xu et al., 2021). Inhibiting the expression of LGMN in prostate cells 22RV1 reduced PI3K but had no effect on the Mammalian target of rapamycin (mTOR) protein (Zhu et al., 2016). Although AKT expression remained unchanged after LGMN elimination, AKT phosphorylation was drastically reduced. As a result of stimulating the PI3K/AKT signaling pathway, LGMN may have a crucial role in the proliferation, invasion, and survival of cancer cells.

### 4.3 LGMN participates in the integrin signaling pathway

A class of heterodimer surface receptors known as integrins can be expressed by blood arteries linked with tumors. Many malignant malignancies have abnormally high levels of LGMN and v integrin in their tumor cells and angiogenic systems. Integrins are expressed by a wide range of cell types, from fibroblasts and endodermal cells to immune cells, and can send signals to govern these cells’ development, proliferation, development, and adhesion (Shen et al., 2020). Breast cancer cell-derived LGMN has an RGD motif that can interact with endothelium integrin v3 to cause v3 to shut. Through the STAT3 signaling pathway, integrin v3 closure indirectly suppresses ZO-1 expression and ultimately raises endothelial cell permeability, promoting the spread of tumor cells. Ovarian cancer is epithelial ovarian cancer (EOC). Several integrins, including 51, 21, and v3 (Deng et al., 2012; Naci et al., 2015), were selectively expressed. Particularly, the proliferation and migration of human peritoneal mesothelial cells were impacted by the 51/LGMN complex in exosomes released by EOC cells (HPMC). 51 and LGMN are both significantly expressed and co-localized in the EOC and HPMC at the same time. Additionally, alterations in the FAK/AKT/Erk of the 51/LGMN downstream pathway and variations in EMT protein level are brought about by co-culturing HPMC with EOC-derived exosomes.

### 4.4 LGMN participates in the TGF-β signaling mechanism

Legumain (Lgmn) is highly expressed in lung tissues and mainly localized in macrophages. The deposition of extracellular matrix proteins around pulmonary vessels caused by Lgmn is closely related to the activation of transforming growth factor-β1 (TGF-β1), and LGMN can activate matrix metalloproteinase-2 (MMP-2) The vertebrate TGF-superfamily, which is made up of TGF-activin and bone morphogenetic protein, is a sizable family of TGFs. Mammalian TGFs come in three varieties: TGF-1, TGF-2, and TGF-3. Smads are related to the Sma proteins found in nematodes and *drosophila*. TGF-receptor II (TRII), which it binds to, causes intracellular signal transduction to be activated. Smad2 and Smad3 are phosphorylated by TGF-1 activating TGF-receptor type I (TRI) kinase. Starting Smad2 and Smad3 then combine with Smad4 to create oligomeric complexes. To control the transcription of their target genes, these oligomeric complexes enter the nucleus (Conidi et al., 2011; Meng et al., 2013). To overcome EMT-related drug resistance, was investigated the TGF-3/FAK pathway targeting lipid metabolism and used LGMN activation to accomplish tumor-related macrophage repolarization (Wang et al., 2022).

### 4.5 LGMN participates in the P53 signaling pathway

P53 is a tumor suppressor motif. It was analyzed DJ-1-knockout cells and found that P53 could regulate the expression and protein enzyme activity of LGMN through DJ-1 in mice. It has been found that most tumor types have P53 missense mutations, but in most glioblastomas, P53 mutations are rare. In 2020, found a new method to regulate the activity of P53 protein (Peng et al., 2018). Activated LGMN would cut P53 protein at N311 to produce P53 fragments, which lost the function of inhibiting oncogene transfer. Thus, glioblasts’ tumor genesis, proliferation, and anti-apoptotic activity were indirectly promoted.

## 5 Relationship between LGMN and tumor microenvironment

**FIGURE 1:**
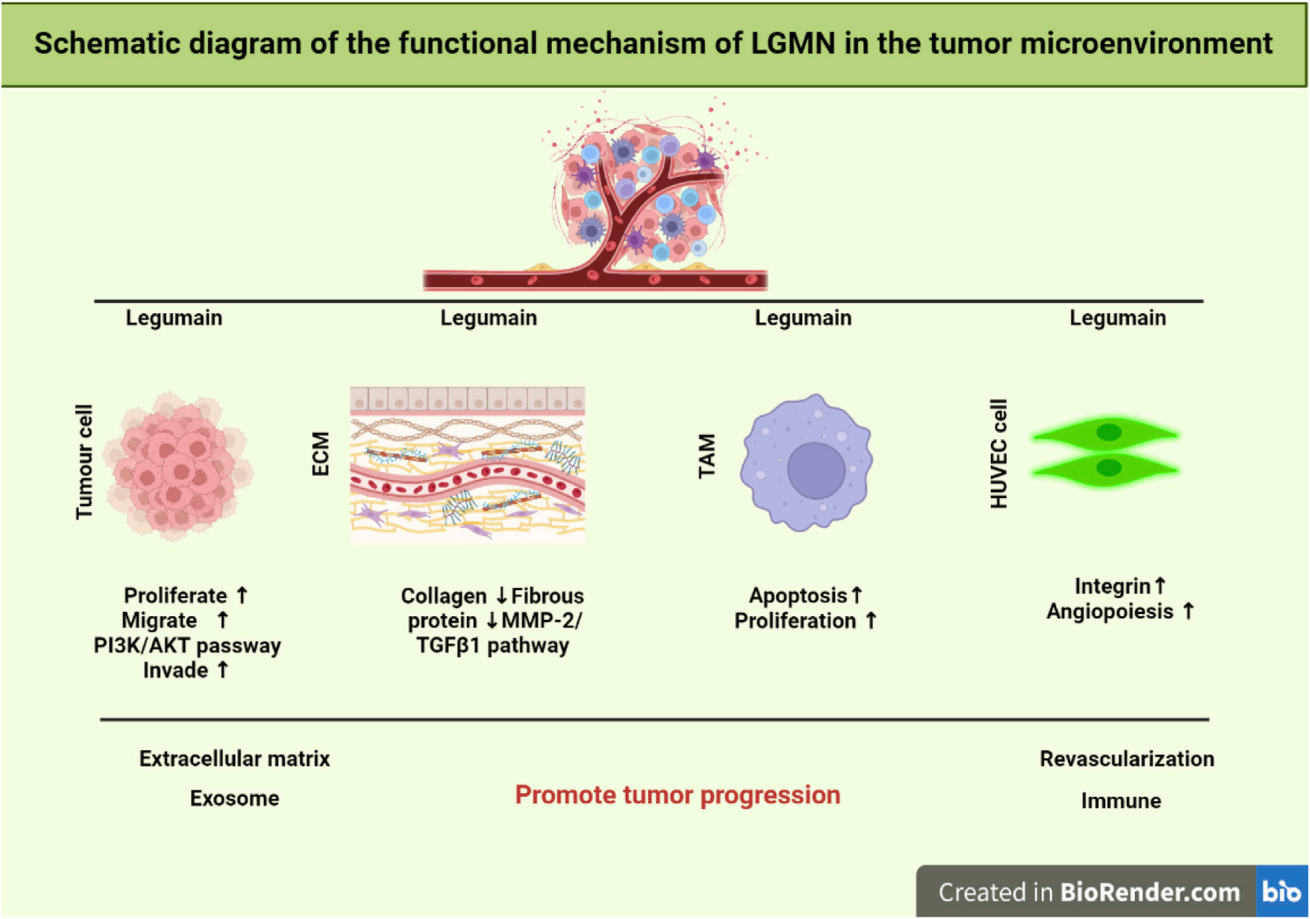
Schematic representation of LGMN functional mechanism in the tumor microenvironment. It was found that the TGF-3/FAK pathway targets lipid metabolism to overcome the treatment resistance related to EMT. LGMN activation is exploited to induce TAM repolarization. Whereas cellular proliferation, invasion, and metastasis are all regulated by the PI3K/AKT pathway. Finally, lowering TAM levels in the tumor stroma can drastically modify the tumor microenvironment, hence limiting tumor development and spreading.

The development and spread of tumors depend on the tumor microenvironment (TME). It is mostly composed of cells that are themselves tumors, as well as macrophages and fibroblasts that are linked with tumors, vascular endothelial cells, matrix proteins, T lymphocytes, and B lymphocytes. Many non-tumor cells are attracted to or activated by tumor cells in TME, and these cells then affect the microenvironment in one of two ways: directly, by the release of factors, or indirectly, through the induction of tissue hypoxia or necrosis (Zhang et al., 2013) (Figure 1).

### 5.1 LGMN affects the microenvironment of angiogenesis

LGMN has highly expressed in neovascularization endothelial cells in the tumor microenvironment. To study the effect of LGMN on the lumen formation of human venous endothelial cells (HUVEC), it was added APEI, an inhibitor of LGMN, into the medium (Ding et al., 2022; Lu et al., 2022). The experimental results show that the number of intact tubules formed by HUVEC cells was significantly reduced and further decreased with the increase of APEI addition, suggesting that the high expression of LGMN in vascular endothelial cells of tumor tissue may promote the formation of tumor neovascularization, improve tumor blood supply, and promote tumor growth. Moreover, LGMN has been confirmed to be significantly associated with tumor neovascularization and tumor-associated macrophages. Double-stained tumor neovascularization with CD31 and LGMN and found that LGMN was highly expressed in endothelial cells with the proliferation of neovascularization through flow cytometry analysis (Castro Jaramillo, 2020).

### 5.2 LGMN affects tumor-associated macrophages

Tumor-associated macrophages (TAMs) are vital components of the tumor microenvironment and belong to M2-type macrophages. TAMs promote tumor matrix remodeling, neovascularization, tumor cell proliferation, and metastasis by secreting various growth factors, angiogenic factors, and proteases. Some myeloid cells are derived from monocytes that differentiate into TAMs after exposure to the local tumor microenvironment. It was constructed a mouse model of breast cancer with LGMN gene deletion by knocking out the LGMN gene in TAMs and found that tumor growth was significantly inhibited. Detecting isolated primary tumor cells demonstrated that LGMN knockout in TAMs inhibited tumor growth, proliferation, and apoptosis. Additionally, compared to the control group, the phosphorylation of p38MAPK, a significant upstream pathway involved in the activation of aging, was increased in isolated primary tumor cells and Lgmn −/− mouse tumor tissues. These findings imply that LGMN deletion in TAMs can greatly accelerate tumor cell aging *in vivo*. The elevated expression of LGMN in TAMs of colorectal cancer tissue samples. Secondly, LGMN knockdown TAMs were shown to be able to prevent tumor growth *in vivo* by comparing the effects of LGMN overexpression TAMs and LGMN knockdown TAMs.The expression of angiogenic marker CD31 was significantly inhibited by immunohistochemistry (Xu et al., 2022), and the expression of malignant growth marker Ki67 was down-regulated in TAM-related tumors inhibited by LGMN, suggesting that LGMN-inhibited TAM can reduce tumor growth and angiogenesis *in vivo*. In conclusion, reducing the amount of TAM in the tumor matrix can alter the tumor microenvironment, thus significantly inhibiting tumor growth and metastasis.

### 5.3 LGMN affects the formation of extracellular matrix

The extracellular matrix (ECM) comprises polysaccharides, proteins, or proteoglycans, which are synthesized by cells and distributed between cells or on the surface of cells. The extracellular matrix can not only support and connect but also regulate the physiological functions of tissues and cells, playing a crucial role in tumor formation and metastasis, as well as in cell proliferation, differentiation, and maintenance of tissue homeostasis (Mohan et al., 2020). LGMN can be secreted into TME to reshape ECM. it was showed co-localization of LGMN and fibronectin in xenografted tumor tissues by immunofluorescence staining, indicating that LGMN could be secreted into TME, and LGMN could lead to catabolic metabolism of the main components of ECM, fibrin and collagen I, thus playing a role in remodeling ECM. it was proved by Western blot that LGMN expression significantly increased collagen, fibrin, and elastin expression levels in human aortic smooth muscle cells (Ozawa et al., 2019). It was showed that LGMN increased the synthesis of ECM protein by activating MMP-2/transforming growth factor-β1 signal transduction in pulmonary arterial VSMC in a mouse model of pulmonary hypertension (Bai et al., 2019).

### 5.4 LGMN affects the function of exosomes

Exosomes are vesicles composed of a bilayer of phospholipids and are secreted by live cells. Their size ranges from 30 to 150 nm. They develop from the endocytosis cavity. Neoplastic cell-derived exosomes successfully transport miRNA, mRNA, and protein to other cells, which controls cell proliferation, invasion, and angiogenesis (Gao et al., 2014; Jeong et al., 2014; Kalluri and LeBleu, 2020). Exosomes rich in LGMN have been shown to promote the proliferation, migration, and invasion of pancreatic ductal adenocarcinoma cells. Exosomes lacking LGMN, on the other hand, decreased the capacity of cancer cells to invade. Stable AEP-KD SKOV3 cell lines in order to investigate the role of exosomes harboring LGMN. They verified that exosome-based LGMN complex inhibition might suppress HPMC migration and proliferation. Analysis of clinical samples revealed that individuals with advanced epithelial ovarian cancer had elevated levels of LGMN complex expression in both serological and ascites-derived exosomes. The findings further imply that organ-specific cells can prepare pre-metastatic states by ingesting tumor-derived exosomes (Hoshino et al., 2015). Therefore, LGMN may be crucial for the development of some malignancies in ways that depend on exosomes.

**FIGURE 2:**
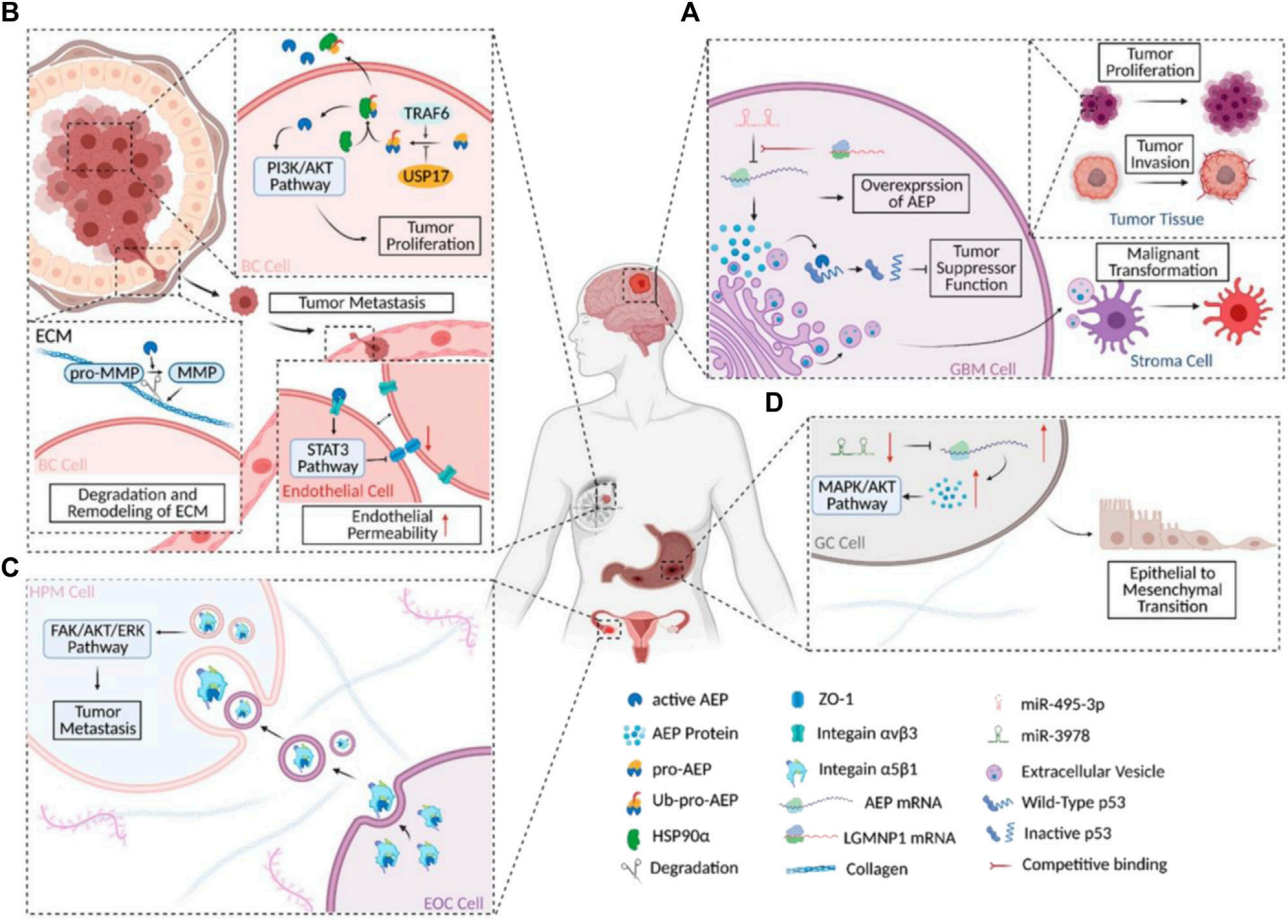
AEP’s functions in different carcinomas schematically. **(A)** AEP’s biochemical and regulatory mechanism in GBM. **(B)** AEP biochemistry and regulation in BC. **(C)** A simplified diagram depicting the molecular process and signaling pathway(s) involved in AEP’s action in EOC. **(D)** A simplified view of the signaling cascade and regulatory mechanism at work during AEP regulation in GC. this figure was reproduced under common creative licenses (Zhang and Lin, 2021).

It was claimed in 2020 that scientists had discovered an entirely new method of controlling the P53 protein’s function. Glioblastoma (GBM) cells’ ability to grow, proliferate, and inhibit apoptosis is indirectly aided by activated AEP’s (Legumain) cleavage of the P53 protein at position 311 (N311). This process produces P53 fragments that are devoid of the protein’s ability to repress oncogene transcription. AEP’s function in GBM is intriguing because it is not confined to tumor cells alone. Activated AEP was released by GBM cells into the TME *via* extracellular carriers (EVs). The release of EVs, which are membrane-bound nanoparticles, allows all cells to participate in a shared system of intercellular communication. The stromal cells in GBM tumor microenvironments (TME) are called astrocytes (Balkwill et al., 2012), and EVs with high levels of AEP promote the malignant transition of non-reactive astrocytes into inflammatory cells (Figure 2).

The malignant alteration of astrocytes by AEP (Lin et al., 2020b) could be indirectly demonstrated by another investigation on cell signaling and GBM invasiveness. Suppression of the P53 pathway and regulation of the MYC pathway are shown to be the mechanisms by which GBM-EV-exposed normal astrocytes acquire a malignant phenotype. However, the primary components of EVs were not investigated in this study, and it is plausible that AEP inhibits the P53 pathway and activates the MYC system in normal astrocytes, resulting in a reactive oncogenic that boosts GBM cells’ tumorigenicity. Additional research is required to support this hypothesis (Broekman et al., 2018; Hallal et al., 2019).

## 6 Application prospect of LGMN as a target in tumor immunotherapy

Targeting LGMN may be a promising approach for creating tumor immunotherapy because LGMN plays a crucial role in the immune system and is preferentially over expressed in tumor microenvironments and tumor tissues (Liu et al., 2003).

### 6.1 DNA vaccines based on LGMN

DNA vaccines based on LGMN were developed to check efficiently protected BALB/c mice from tumor cell attack by generating a specific CD8^+^ T cell response against TAMs with high expression of LGMN (Castro Jaramillo, 2020). In addition to using a polypeptide vaccination to create anti-PD-1 antibodies as a combination therapy to reverse immunosuppression in the tumor microenvironment, utilised the evidence from the LGMN monopeptide DNA vaccine (Karyampudi et al., 2014). The combination approach increased the vaccine’s effectiveness, caused tumor regression, encouraged CD8^+^ T cell invasion during tumor regression, and increased the median survival time of tumor-bearing animals. Before DNA vaccines may be used in the clinic, more research must be done on the intricate host immunological response they induce.

### 6.2 Inhibition of LGMN by small molecule inhibitors

SAR131675 is a tyrosine kinase inhibitor. That SAR131675 can induce practical anti-tumor effects by targeting several *in situ* and isogenic breast tumor models to reduce drench invasion and avoid lung metastasis (Alam et al., 2012). In addition, treatment with SAR131675 could reduce the number of macrophages that expressed LGMN at a high level and were immunocompromised in the tumor microenvironment (Espagnolle et al., 2014). These results suggest that tyrosine kinase inhibitors such as SAR131675 have the potential to target LGMN in the tumor microenvironment.

### 6.3 LGMN activation prodrug

Due to the high expression of LGMN in tumor tissues, the precursor drugs specifically activated by LGMN may be selective and tumor-specific. This mechanism may reduce the incidence of non-specifically targeted drug adverse reactions. To minimize the side effects of the chemotherapy drug doxorubicin (Dox), DoX-based tumor-specific LGMN activation prodrug Legubicin. Legubicin was well tolerated in tumor mice with high LGMN hypertrophic levels, showing higher efficacy and a reduced incidence of weight loss compared to similar mice treated with adriamycin.

### 6.4 Azopeptide epoxide pair

Inhibition of LGMN Azopeptide is a peptide replaced by one or more ammoniac acid residues. It was designed azopeptide epoxide to inhibit LGMN in Schistosoma mansoni and porcine kidney irreversibly (James et al., 2003). The designed azopeptide epoxides are specific for LGMN but not for other proteases. In addition, compounds with potentially irreversible interactions with LGMN may interfere with the physiological function and protease activity of LGMN.

### 6.5 DNA-mutated vaccine

Injecting MK 16 cells into C57BL/6 mice, a DNA vaccine targeting LGMN was proven to be efficacious in 2014 (Smahel et al., 2014). The DNA vaccine’s efficacy was improved by introducing a mutation to the RGD motif, which led to the formation of a targeted nanoparticle that inhibited LGMN development and hampered its cellular localization. Further improvement in immunogenicity was achieved by introducing a p30 auxiliary epitope from tetanus toxin in addition to the alteration in the RGD motif. Although the improved performance of the modified vaccination has been observed, further research is needed to fortify this strategy.

**FIGURE 3:**
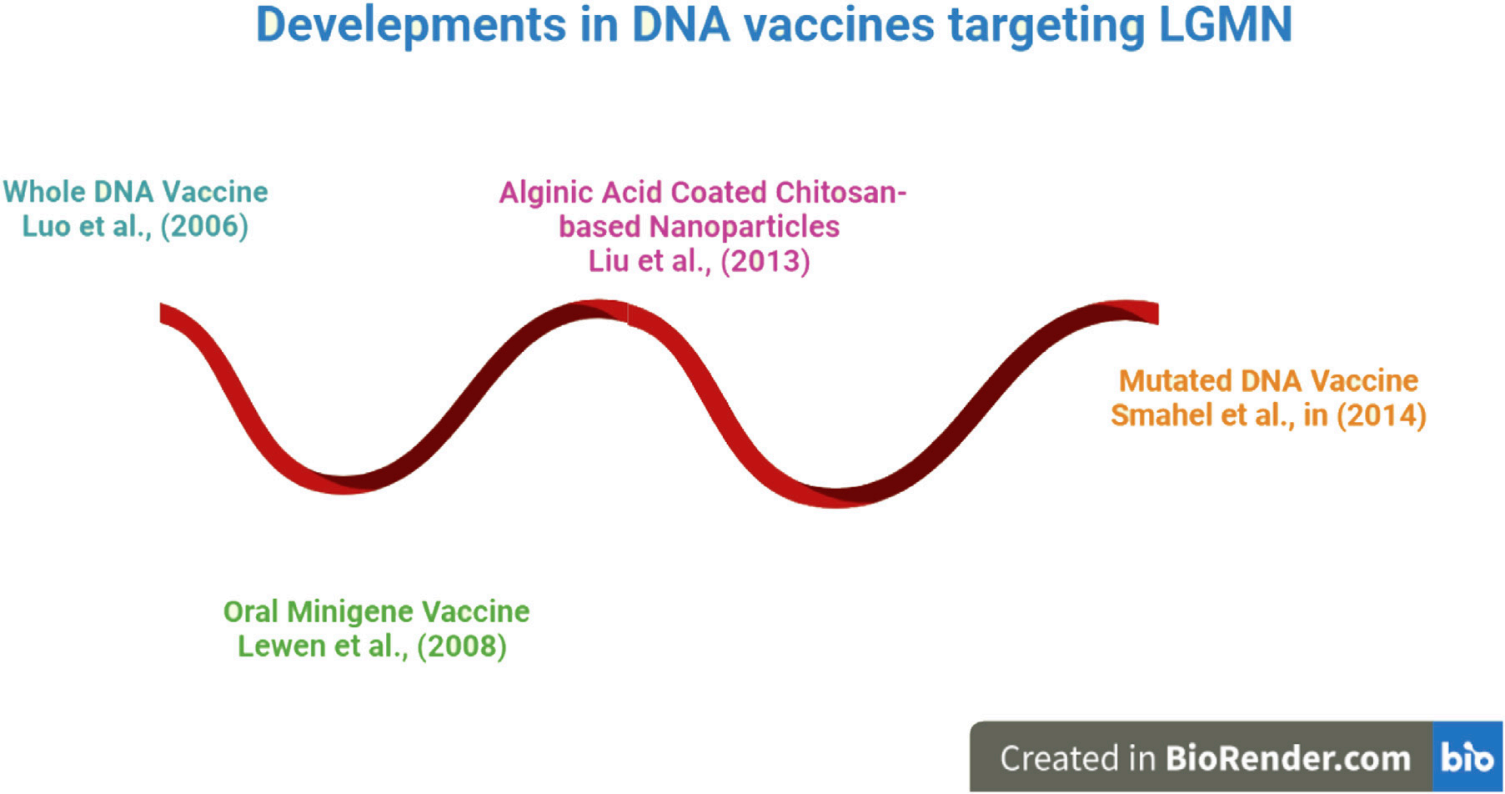
An Overview of DNA Vaccines against LGMN. Research has shown that work on DNA vaccinations began in earnest in 2006. DNA vaccines have been engineered in a variety of forms, from whole DNA to altered DNA enclosed in a nanoparticle. Later on! DNA vaccines experienced got tremendous development, proven to be successful at lowering tumor burden and concentrating on LGMN.

The evolution of DNA vaccines is depicted beginning in 2006 in Figure 3. DNA vaccines have been engineered in a variety of forms, from encapsulated nanoparticles containing altered DNA to those made up entirely of intact DNA. DNA vaccines directed against LGMN were shown to be effective in an animal study. The immunization suppressed several oncogenic cytokines and tumor density (Luo et al., 2006). In light of these findings, more research was done, and oral minigene vaccines were developed. Unlike whole-DNA vaccinations, these are more stable and resistant to mutations. Liu et al. (2013) developed vaccinations based on alginic acid and chitosan for easier and more effective delivery; these vaccines showed promise in animal tumor models. As a result of this breakthrough, the creation of a nanoparticle-based Mutant DNA vaccine improved the reliability and effectiveness of DNA vaccines. DNA vaccines, which have recently undergone significant development, have been demonstrated to be effective at reducing tumor burden and focusing on LGMN. These findings have not been tested in human clinical trials and have only been observed in animal models. Because of the vaccines’ apparent efficacy in animal models, researchers now have reason to believe that more effective vaccine candidates targeting LGMN can be developed for human use.

## 7 Suitability of LGMN for imaging

For the purpose of imaging LGMN-expressing cells *in vivo*, Lee and Bogyo (2010) created activity-based probes labeled with near-infrared fluorophores (NIRF). According to the research, azapeptidyl ASN epoxide, which can be converted into a cancer-detecting near-infrared fluorophore, specifically labels LGMN. Porous silicon (PSi) nanoparticles were independently developed, and Y-shaped and single new peptides that specifically target LGMN were encapsulated within them (Kanathasan et al., 2017). More research is required to understand the relationship between PSi-based nanotechnology and breast cancer cells.

### 7.1 Legumain nanobubbles for cancer by ultrasound imaging

Ultrasound (US) imaging, a frequent diagnostic imaging method, has many advantages, including low cost, biosafety, superior temporal, and spatial resolutions, and simplicity of use. One Due to the difference in acoustic impedance between gas and tissue, ultrasonic contrast agents (UCAs), primarily gas-filled echogenic microbubbles (MBs), can significantly improve the quality of ultrasound (US) imaging (Sboros, 2008). On the basis of molecular US imaging, targeted molecular ultrasonic contrast agents (UCAs) have recently been developed for tumor identification. These UCAs are composed of microbubbles that have had their ligands changed to target molecules that are abundantly expressed in tumor technology in recent years (Köse et al., 2020). After intravenous administration, the specific binding of the marker molecules to the ligands enables MBs to accumulate preferentially in tumor vascular, identifying the tumor site.

Legumain, a cysteine protease, has been found to be elevated in a variety of solid tumor types, such as breast, lung, gastric, colon, prostate, lymphoma, ovarian, etc., (Liu et al., 2014; Dall and Brandstetter, 2016). Tumor-associated macrophages (TAMs), which are believed to be primarily present, extremely active, as well as being overexpressed in tumor cells, mediate the tumor microenvironment. Small (49 nm) nanobubbles were synthesized using successive sonication to generate tumor and neovascular “dual targeted”-molecular UCAs, and the NB surface was modified with either AAN or the v3 integrin targeting tripeptide arginine-glycine-aspartic acid (RGD) (Wang et al., 2012; Shen et al., 2020). RGD-modified NBs can preferentially link with v3 integrin in the tumor neovasculature using this integrated method, whereas AAN-modified NBs may promote NB adsorption on tumor cells and tumor-associated macrophages. The dual-targeted NBs that have been created should be quite good at detecting cancers (Dall and Brandstetter, 2016; Mi et al., 2022).

### 7.2 Legumain nanobubbles for cancer by MRI imaging

It is advantageous to foresee its natural substrates to screen for legumain specificity in the prime area. However, the analysis takes longer and requires more work when label-free peptides are employed because they don't offer a direct readout. Internally quenched fluorescence (IQF) substrates are an alternate approach where the peptide sequence is sandwiched between a donor (fluorophore) and an acceptor (quencher). The fluorophore is no longer quenched and increased fluorescence is produced once a peptide link has been hydrolyzed. After then, the fluorophore and quencher are split apart. In 2016, researchers described the development and biological analysis of two IQF substrates with the Lys (Dnp) acceptor and MCA or ACC fluorophores (Poreba et al., 2017; Li et al., 2019). This research highlights the importance of IQF substrates in legumain specificity profiling for the prime areas, when conventional peptideAMC/ACC substrates cannot be employed. Two fluorophores can be combined into a single substrate molecule, with one of the fluorophores acting as a quencher, to create internally quenched fluorescent substrates. Along with this IQF probe, the authors also created an MRI contrast agent using a peptide that has been broken down by the legumain and a complexed form of the metal gadolinium (Gd3+). The Gd-containing portion of this substrate-like probe has the ability to bind to human serum albumin (HSA) and form macromolecules, which would enhance the MRI signal (Chen et al., 2014). Since this probe was able to detect legumain activity in animals bearing CT26 xenografts, the researchers think it may be used to image other proteases in different tumors *in vivo*.

## 8 Conclusion

LGMN has been described as a promising therapeutic and predictive target, and there is mounting evidence pointing to its pivotal involvement in tumour progression and invasion. In light of this, researchers are currently focusing on either creating therapies specifically targeting LGMN or creating new chemotherapeutic drugs that utilize LGMN substrate specificity. Due to the low pH present in the tumor microenvironment, legume proteases are able to efficiently catalyze their targets. However, as legume proteases can drive matrix modification and metastatic dissemination, the absence of native legume inhibitors ultimately leads to cancer progression. LGMN is a cysteine protease that is produced as part of a specific intracellular stress response. It plays a critical role in the development of malignancies by regulating the tumor microenvironment. Some of the key mechanisms include exosome trafficking, regulation of protease activity, regulation of integrin interaction, interference with signaling pathways, and so on. But in many ways, the process is not particularly obvious. Questions remain, however, about how exactly LGMN affects exosomes, how the activation of the PI3K/AKT communicating pathway contributes to the biological effects of LGMN, and how LGMN’s signaling regulation pathway affects certain malignancies. The development of gene vaccinations or inhibitors that specifically target LGMN to suppress the expansion of cancer cells *in vivo* could pave the way for a novel strategy in cancer gene therapy. Although the transfer to clinical therapeutic application is in its early stages, thorough population investigations and mechanism technical study are needed to support and verify its dependability.

In conclusion, LGMN’s regulation route varies greatly across tumor types, microenvironments, and tissues. The keys and challenges for a paradigm shift in this subject lie in a more thorough comprehension and reliability test of this diversity, as well as an investigation of the mechanism that regulates it. In the future, efforts should be directed not only toward the production of efficient legumain inhibitors, but also toward the use of legumain for *in vivo* tumor imaging and therapy, both of which can be targeted in conjunction with legumain to achieve superior therapeutic results.
